# Choline Glycerophospholipid-Derived Prostaglandins Attenuate TNFα Gene Expression in Macrophages via a cPLA_2_α/COX-1 Pathway

**DOI:** 10.3390/cells10020447

**Published:** 2021-02-19

**Authors:** Alma M. Astudillo, Juan P. Rodríguez, Carlos Guijas, Julio M. Rubio, María A. Balboa, Jesús Balsinde

**Affiliations:** 1 Instituto de Biología y Genética Molecular, Consejo Superior de Investigaciones Científicas (CSIC), 47003 Valladolid, Spain; alma@ibgm.uva.es (A.M.A.); rodriguezcasco@me.com (J.P.R.); cguijas@ibgm.uva.es (C.G.); jrubio@ibgm.uva.es (J.M.R.); mbalboa@ibgm.uva.es (M.A.B.); 2 Centro de Investigación Biomédica en Red de Diabetes y Enfermedades Metabólicas Asociadas (CIBERDEM), 28029 Madrid, Spain; 3 Laboratorio de Investigaciones Bioquímicas de la Facultad de Medicina (LIBIM), Instituto de Química Básica y Aplicada del Nordeste Argentino (IQUIBA-NEA), Universidad Nacional del Nordeste, Consejo Nacional de Investigaciones Científicas y Técnicas (UNNE-CONICET), Corrientes 3400, Argentina

**Keywords:** arachidonic acid, eicosanoids, phospholipid remodeling, phospholipase A_2_, inflammation, monocytes/macrophages

## Abstract

Macrophages are professional antigen presenting cells with intense phagocytic activity, strategically distributed in tissues and cavities. These cells are capable of responding to a wide variety of innate inflammatory stimuli, many of which are signaled by lipid mediators. The distribution of arachidonic acid (AA) among glycerophospholipids and its subsequent release and conversion into eicosanoids in response to inflammatory stimuli such as zymosan, constitutes one of the most studied models. In this work, we used liquid and/or gas chromatography coupled to mass spectrometry to study the changes in the levels of membrane glycerophospholipids of mouse peritoneal macrophages and the implication of group IVA cytosolic phospholipase A_2_ (cPLA_2_α) in the process. In the experimental model used, we observed that the acute response of macrophages to zymosan stimulation involves solely the cyclooxygenase-1 (COX-1), which mediates the rapid synthesis of prostaglandins E_2_ and I_2_. Using pharmacological inhibition and antisense inhibition approaches, we established that cPLA_2_α is the enzyme responsible for AA mobilization. Zymosan stimulation strongly induced the hydrolysis of AA-containing choline glycerophospholipids (PC) and a unique phosphatidylinositol (PI) species, while the ethanolamine-containing glycerophospholipids remained constant or slightly increased. Double-labeling experiments with ^3^H- and ^14^C-labeled arachidonate unambiguously demonstrated that PC is the major, if not the exclusive source, of AA for prostaglandin E_2_ production, while both PC and PI appeared to contribute to prostaglandin I_2_ synthesis. Importantly, in this work we also show that the COX-1-derived prostaglandins produced during the early steps of macrophage activation restrict tumor necrosis factor-α production. Collectively, these findings suggest new approaches and targets to the selective inhibition of lipid mediator production in response to fungal infection.

## 1. Introduction

When infections caused by viruses, bacteria or fungi occur, macrophages, strategically infiltrated in all tissues, respond by assembling a sequenced and coordinated set of responses to remove the pathogenic agents. Although macrophages are professional antigen presenting cells for T lymphocytes, prior to the immune synapse they also release cytokines and lipid mediators that extensively regulate the progress of inflammation and subsequently tissue remodeling and repair [1,2,3]. Eicosanoids include a class of lipid mediators derived from the metabolism of polyunsaturated fatty acids such as arachidonic acid (AA) by cyclooxygenases (COX), lipoxygenases, cytochrome P450, or non-enzymatic pathways. Among them, prostaglandins (PG) are bioactive signaling molecules derived from COX and the subsequent activity of terminal synthases on AA. Although PG biosynthesis involves several steps catalyzed by different enzymes, an important regulatory step is the hydrolysis of AA from glycerophospholipids by phospholipase A_2_ enzymes [4,5,6,7].

Zymosan particles are homogenates of *Saccharomyces cerevisiae* cell walls that have been used extensively as a model to study innate immune responses to fungal infections [8]. Although the recognition of molecular patterns associated with fungal pathogens (through pattern recognition receptors) [8,9], the triggering of phagocytosis [10,11], and the production of macrophage-derived cytokines [12,13] were described in great detail in in vivo or in vitro experimental models, less attention has been given to the study of the distribution of eicosanoid precursor fatty acids such as AA between the different membrane glycerophospholipid species [14,15], and their importance in the regulation of eicosanoid production in the early stages of fungal infection. 

In previous work from our laboratory, we analyzed the cellular and molecular events triggered after the stimulation of mouse peritoneal macrophages with zymosan, either native or opsonized, focusing on the phagocytosis process and the associated lipid signaling pathways [15,16,17]. We demonstrated the involvement of several phospholipase A_2_ forms, each acting on distinct phospholipid pools and releasing different fatty acids [15,18,19]. Also, we showed the importance of coenzyme A-independent transacylation reactions to shape the cellular AA pools and their involvement in some macrophage responses [20,21,22]. In the current work we have utilized advanced mass spectrometry-based lipidomic approaches to characterize the immediate generation of PG and time-dependent changes of AA-containing phospholipid species in zymosan-stimulated mouse peritoneal macrophages. Using pharmacological inhibitors and antisense oligonucleotide approaches, we determined the central role that group IVA cytosolic phospholipase A_2_ (cPLA_2_α) plays in generating free AA substrate for PG production. Importantly, our data provide evidence that immediate PG production arises from the hydrolysis of choline-containing glycerophospholipids (PC) and phosphatidylinositol (PI) pools, with no clear involvement of ethanolamine-containing glycerophospholipids (PE). Our work also shows that PG levels negatively modulate the expression on tumor necrosis factor-α (TNFα) levels in activated macrophages, thus suggesting that the cPLA_2_α/COX-1 pathway regulates the execution of early proinflammatory responses by the macrophages.

## 2. Materials and Methods 

### 2.1. Reagents

Cell culture medium was from Molecular Probes-Invitrogen (Carlsbad, CA, USA). Organic solvents (Optima^®^ LC/MS grade) were from Fisher Scientific (Madrid, Spain). Lipid standards were from Avanti (Alabaster, AL, USA) or Cayman (Ann Arbor, MI, USA). Silicagel G thin-layer chromatography plates were from Macherey-Nagel (Düren, Germany). [5,6,8,9,11,12,14,15^−3^H]Arachidonic acid (180 Ci/mmol) and [1^−14^C]arachidonic acid (50 µCi/mmol) were from PerkinElmer (Boston, MA, USA). Inhibitors were from Cayman. All other reagents were from Sigma-Aldrich (Madrid, Spain).

### 2.2. Cell Culture and Stimulation Conditions

Resident peritoneal macrophages from Swiss male mice (University of Valladolid Animal House, 10–12 weeks old) were obtained by peritoneal lavage using 5 mL cold phosphate-buffered saline, and cultured in RPMI 1640 medium with 10% heat-inactivated calf serum, 100 U/mL penicillin, and 100 μg/mL streptomycin, as described elsewhere [23]. For antisense inhibition experiments, RAW264.7 macrophage-like cells were used. These cells were grown in Dulbecco’s modified Eagle’s medium supplemented with 10% (*v/v*) fetal bovine serum, 100 U/mL penicillin, 100 µg/mL streptomycin, and 2 mM L-glutamine at 37 °C in a humidified atmosphere of 5% CO_2_ at 37 °C, as previously described [24,25]. Zymosan was prepared as described [26]. Only zymosan batches that demonstrated no measurable endogenous phospholipase A_2_ activity, as measured by in vitro assay under different conditions [27,28,29,30], were used in this study. Cell protein was measured using a commercial kit (BioRad, Hercules, CA, USA). 

For labeling of the cells with [^3^H]AA and [^14^C]AA, they were first labeled with 0.25 µCi/mL [^3^H]AA for 20 h and then with 0.1 µCi/mL [^14^C]AA for 30 min. Labeled AA that had not been incorporated into cellular lipids was removed by washing the cells four times with serum-free medium containing 0.5 mg/mL albumin. After the stimulations, the supernatants were acidified to pH 3.5 with 5 M formic acid and extracted with isopropanol/diethyl ether (1:1.5, *v/v*), and radiolabeled prostaglandins and free AA were separated by thin-layer chromatography, using ethyl acetate/acetone/acetic acid (90:5:1) as the mobile phase [31]. The cell monolayers were homogenized and the lipids were extracted according to Bligh and Dyer [32]. For separation of phospholipid classes, plates were run twice with chloroform/methanol/28% (*w/w*) ammonium hydroxide (60:37.5:4, *v/v/v*) as the mobile phase, using plates impregnated with boric acid [33]. The bands corresponding to the different lipid classes were scraped from the plates and their radioactive content was determined by scintillation counting using a Beckman Coulter LS6500 Liquid Scintillation Counter (Beckman, Fullerton, CA, USA).

### 2.3. Liquid Chromatography/Mass Spectrometry (LC/MS) Analyses of Prostaglandins

Analysis of prostaglandins by LC/MS was carried out exactly as described elsewhere [15,33], using an Agilent 1260 Infinity high-performance liquid chromatograph equipped with an Agilent G1311C quaternary pump and an Agilent G1329B Autosampler, coupled to an API2000 triple quadrupole mass spectrometer (Applied Biosystems, Carlsbad, CA, USA). Quantification was carried out by integrating the chromatographic peaks of each species and comparing with a calibration curve made with analytical standards.

### 2.4. Liquid Chromatography/Mass Spectrometry (LC/MS) Analyses of Phospholipids

This was carried out exactly as described elsewhere [15,22,34,35], using a high-performance liquid chromatograph equipped with a binary pump Hitachi LaChrom Elite L-2130 and a Hitachi Autosampler L-2200 (Merck, Madrid, Spain), coupled on-line to a Bruker Esquire 6000 ion-trap mass spectrometer (Bruker Daltonics, Bremen, Germany). Phospholipid molecular species were identified by multiple reaction monitoring experiments on chromatographic effluent by comparison with previously published data [15,22,34,35]. 

### 2.5. Gas chromatography/Mass Spectrometry (GC/MS) Analyses

Total lipids from approximately 10^7^ cells were extracted according to Bligh and Dyer [32], and internal standards were added. Phospholipids were separated from neutral lipids by thin-layer chromatography, using *n*-hexane/diethyl ether/acetic acid (70:30:1, *v/v/v*) as the mobile phase [36]. The phospholipid bands were scraped from the plate, and fatty acid methyl esters were obtained from the various lipid fractions by transmethylation with 0.5 M KOH in methanol for 60 min at 37 °C [37,38,39,40]. Analysis was carried out using an Agilent 7890A gas chromatograph coupled to an Agilent 5975C mass-selective detector operated in electron impact mode (EI, 70 eV), equipped with an Agilent 7693 autosampler and an Agilent DB23 column (60 m length × 0.25 mm internal diameter × 0.15 µm film thickness) (Agilent Technologies, Santa Clara, CA, USA). Data analysis was carried out with the Agilent G1701EA MSD Productivity Chemstation software, revision E.02.00 [37,38,39,40].

### 2.6. iPLA_2_β Antisense Inhibition Studies

The iPLA_2_β antisense oligonucleotide used in this study has been described in previous studies from our laboratory [41,42,43,44]. The oligonucleotides used were as follows: antisense, 5′-CTC CTT CAC CCG GAA TGG GT; sense, 5′-ACC CAT TCC GGG TGA AGG AG. Both sense and antisense oligonucleotides contained phosphorothioate linkages to limit degradation. The oligonucleotides were mixed with Lipofectamine RNAiMAX (Thermo Fisher Scientific, Walthman, MA, USA) following the manufacturer’s instructions. Oligonucleotide treatment and culture conditions were not toxic for the cells as assessed by trypan blue dye exclusion and by quantitating cellular protein.

### 2.7. Quantitative PCR

Total RNA was extracted from the cells with TRIzol reagent (Invitrogen, Carlsbad, CA, USA) according to the manufacturer’s instructions, and 2 µg RNA was reverse transcribed using random primers and oligo d(T) and Moloney murine leukemia virus reverse transcriptase (Ambion, Austin, TX, USA). Quantitative PCR was carried out with an ABI 7500 machine (Applied Biosystems, Carlsbad, CA, USA) using Brilliant III Ultra-Fast SYBR Green qPCR Master Mix (Agilent Technologies, Santa Clara, CA, USA). Cycling conditions were as follows: 1 cycle at 95 °C for 3 min and 40 cycles at 95 °C for 12 s, 60 °C for 15 s, and 72 °C for 28 s [45]. The replicates were averaged, and fold induction was determined in ΔΔCt-based fold-change calculations, with cyclophilin A as a control [46]. Primer sequences are available upon request.

### 2.8. Data Analysis

The results are shown as means ± standard error of the mean and were analyzed for statistical significance by *t*-test (two groups) or by ANOVA (more than two groups), followed Tukey’s post hoc test, using GraphPad Prism software. A value of *p* < 0.05 was considered statistically significant.

## 3. Results

### 3.1. Immediate Synthesis of Prostaglandins by Macrophages

**Figure 1:**
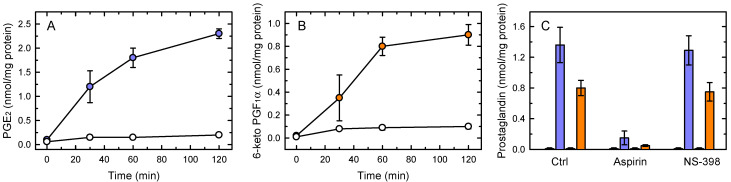
PG production by zymosan-stimulated macrophages. The cells were left untreated (open symbols) or treated with 1 mg/mL zymosan (colored symbols) for the indicated times, and the levels of PGE_2_ (**A**) and 6-keto-PGF_1α_ (**B**) in the supernatants were measured by LC/MS. (**C**) Effect of COX inhibitors on PG production. Aspirin (20 µM), NS-398 (5 µM) or neither (Ctrl) were present at the time the cells were stimulated with zymosan. After 1 h, PGE_2_ (purple bars) and 6-keto-PGF_1α_ (orange bars) were measured by LC/MS. The small bars to the left of the colored bars represent the basal PG production in the absence of stimulus. The results, given as nmol PG per mg of total cell protein, are shown as means ± standard error (n = 6).

Figure 1A shows that acute stimulation of murine resident peritoneal macrophages with yeast-derived zymosan results in the immediate production of PGE_2_ (Figure 1A) and PGI_2_ (recovered as 6-keto-PGF_1α_; Figure 1B). These two species accounted for more than >90% of total AA metabolites produced at these short times. 

Previous studies have demonstrated that COX-1 is constitutively expressed in macrophages, whereas the COX-2 isoform is induced only after at least 2–3 h of cell stimulation [47,48]. In agreement with these observations, PG production was completely blocked by the general inhibitor aspirin, whereas the selective COX-2 inhibitor NS-398 had no discernible effect (Figure 1C). Thus these results demonstrate that immediate PG production in activated macrophages is due to COX-1.

### 3.2. Analysis of the AA Mobilization Response 

**Figure 2:**
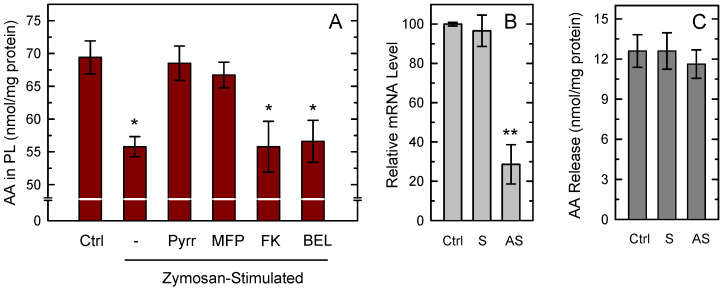
AA mobilization from macrophages. (**A**) The cells were either unstimulated (Ctrl) or stimulated with 1 mg/mL zymosan for 1 h in the absence (-) or presence of the following inhibitors: 1 µM pyrrophenone (Pyrr), 10 µM methyl arachidonyl fluorophosphonate (MFP), 10 µM FKGK18 (FK), or 10 µM bromoenol lactone (BEL). Afterward, total content of AA in phospholipids was measured by GC/MS. Results are shown as means ± standard error of the mean (n = 6). * *p* < 0.05, significance of stimulated cells versus control cells. (**B**) The cells were treated for 36 h with sense (S) or antisense (AS) oligonucleotide, or vehicle (Ctrl), and iPLA_2_β mRNA levels were determined by quantitative PCR. (**C**) After the treatments, the cells were stimulated with zymosan, and AA release was determined by GC/MS. The fatty acid release was calculated by subtracting the amount of phospholipid-bound AA in unstimulated cells from that in stimulated cells. Results, given as nmol AA per mg of total cell protein, are shown as mean values ± standard error of the mean. (n = 4). ** *p* < 0.01, significantly different from control cells.

To analyze the availability of AA substrate for PG synthesis, we measured AA mobilization by the zymosan-stimulated macrophages (Figure 2A). GC/MS analyses of AA content in the macrophages after 1 h of zymosan stimulation indicated that the cells lost 20–25% of their initial AA content, equaling to 10–15 nmol/mg protein, an amount well above what was necessary to sustain the COX-1-dependent PG production shown in Figure 1A. In turn, the data indicated that a substantial part of the released AA remains as unmetabolized free fatty acid. Figure 2A also shows that the zymosan-stimulated response was almost completely prevented by the presence in the incubation medium of the cPLA_2_α inhibitors pyrrophenone [49] and methyl arachidonyl fluorophosphonate (MFP) [50], highlighting the role of cPLA_2_α as the major mediator of the response. MFP is also a potent inhibitor of iPLA_2_β [51]; however the selective inhibitors bromoenol lactone (BEL) [52] and the fluoroketone FKGK18 [53] had no discernible effect, ruling out a significant role for iPLA_2_β in the response (Figure 2A). This conclusion was further supported by experiments utilizing cells deficient in iPLA_2_β by the use of an antisense oligonucleotide which we and others have previously used with success to reduce the expression of this enzyme in a variety of cells [54,55,56]. Since we were unable to find reliable antibodies against murine iPLA_2_β, the efficiency of antisense inhibition was analyzed by determining mRNA levels by qPCR. We detected an mRNA decrease of 60–70% (Figure 2B). iPLA_2_β-deficient cells, however, did not show any significant reduction of their capacity to release AA to the extracellular medium in response to zymosan (Figure 2C), providing additional evidence that the enzyme does not play a significant role in the response.

**Figure 3:**
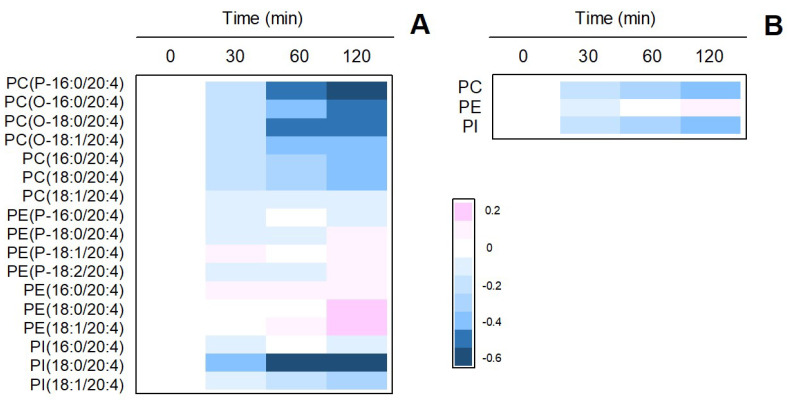
Time-dependent changes of AA-containing phospholipid species in zymosan-stimulated macrophages. (**A**) The cells were stimulated with 1 mg/ml zymosan for the times indicated. The figure shows a heat map of all species identified by LC/MS. (**B**) Sum of AA-containing species within phospholipid classes. The phospholipids are designated according to Fahy et al. [59]. Fatty chains within the different phospholipid species are designated by their number of carbons followed by a colon, and the number of double bonds. A designation of O- before the first fatty chain indicates that the sn-1 position is ether linked, whereas a P- designation indicates a plasmalogen form (sn-1 vinyl ether linkage). Phospholipids containing two ester bonds have no designation. The experiment shown is representative of three independent determinations with duplicate incubations.

Having established the role of cPLA_2_α as the mediator of zymosan-stimulated release, in the next series of experiments we set out to determine whether phospholipid pools existed that selectively accounted for PG production. In the context of this work, the term phospholipid pool refers to each of the AA-containing phospholipid classes present in the cells. In the first place, we determined by LC/MS the time-course of changes in AA-containing phospholipid molecular species upon stimulation with zymosan (Figure 3). All AA-containing PC species experienced marked decreases with time. A single PI species, namely PI(18:0/20:4) also showed strong time-dependent decreases. In contrast, PE species showed little to no decreases and, as a matter of fact, some of the species slightly increased with time. It has to be noted in this regard that, during cell activation, rapid transfer of AA moieties from PC to PE occurs via CoA-independent transacylation reactions [57,58], which obscures the actual contribution of PE species to overall AA mobilization.

**Figure 4:**
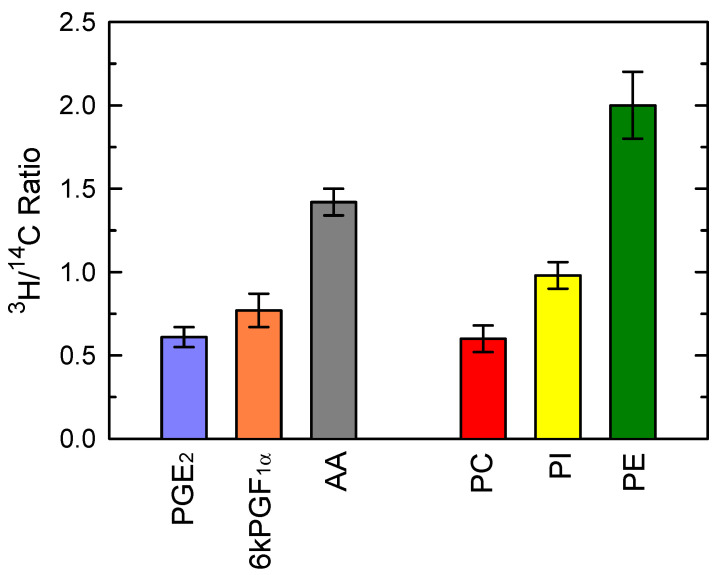
Phospholipid sources of prostaglandins produced by zymosan-stimulated macrophages. The cells, labeled with both [^3^H]AA and [^14^C]AA were treated with 1 mg/mL zymosan for 1 h. Afterward, the ^3^H/^14^C ratio of extracellularly liberated PGE_2_, 6-keto-PGF_1α_ and AA was calculated. The ^3^H/^14^C ratio for AA-containing PC, PI and PE is shown for comparison.

Collectively, the data of Figure 3 point at PC and PI as likely precursors of the AA being used for PG production during zymosan activation of the macrophages. To directly examine this possibility, we took advantage of the fact that the cellular AA phospholipids can be differentiated by double-labeling them with [^3^H]AA and [^14^C]AA at different times. The cells were first labeled with [^3^H]AA for 20 h, a time frame long enough to allow for the radiolabeled fatty acid to equilibrate among phospholipids and thus resemble the endogenous distribution of AA [60]. Under these conditions, the order of incorporation of radiolabel in phospholipid classes was PE > PC ≫ PI. After the 20h incubation period, the cells were pulse-labeled with [^14^C]AA for 30 min. At these short labeling times, the distribution of radiolabeled AA phospholipids dramatically differs from that seen at long incubation times, in that most of the radiolabel is incorporated into PC. PI and PE incorporate much lesser amounts [60]. Subsequent to the double-labeling, the cells were treated with 1 mg/mL zymosan for 1 h, and the ^3^H/^14^C ratios were determined in the phospholipid classes as well as in the prostaglandins and free AA liberated to the incubation medium (Figure 4). The ^3^H/^14^C ratio for PGE_2_ was very close to that of PC, strongly suggesting that PC was indeed the principal originator of the AA being converted to this PG. As for 6-keto-PGF_1_α, its ^3^H/^14^C ratio was intermediate between those of PC and PI, consistent with a contribution of both phospholipid classes. Note in contrast the high ^3^H/^14^C value for free AA, which was intermediate between that of PE and those of PC and PI, suggesting that free AA had been derived from all phospholipid classes (Figure 4).

### 3.3. COX-1-Mediated Prostaglandin Production Regulates TNFα Production

Recent evidence suggests the existence of a regulatory interplay between the production of certain cytokines or chemokines and the synthesis/action of eicosanoids or their receptors. This is exemplified by studies showing that TNFα and PGE_2_ act synergistically to induce IL-8 expression [61], and that IL-10, a cytokine with proven anti-inflammatory functions, contributes to PGE_2_ signaling through the upregulation of the EP4 receptor [62]. Therefore it seemed reasonable to investigate whether a connection existed in our system between early PG production and cytokine expression.

**Figure 5:**
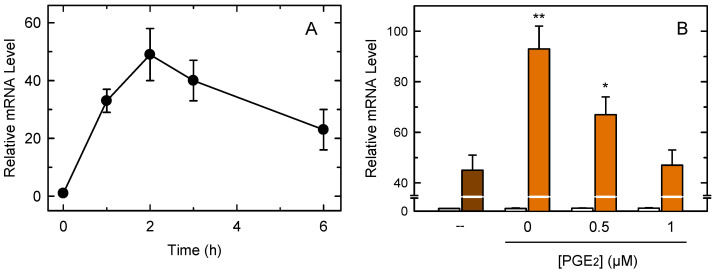
Analysis of Tnf expression in macrophages. (**A**) The cells were treated with 1 mg/mL zymosan for different periods of time. Afterward, Tnf expression was studied by qPCR. (**B**) The cells were stimulated with zymosan in the absence (brown bar) or presence (orange bars) of 20 µM aspirin plus the indicated concentrations of PGE_2_. Open bars denote incubations in the absence of zymosan. After the 1-h incubations, Tnf expression was studied by qPCR. The results, are shown as means ± standard error (n = 6). ** *p* < 0.01 or * *p* < 0.05, significance of incubations in the presence of aspirin versus incubations in the absence of aspirin.

Figure 5A shows that zymosan stimulation of the macrophages induced the expression of *Tnf*, the gene coding for the cytokine TNFα. *Tnf* induction occurred very rapidly, reaching a maximum at 2 h of incubation and decreasing thereafter. Given that the peak of *Tnf* induction occurred within the same time frame as the COX-1-dependent PG production by the macrophages, we sought to analyze the possibility of whether the two events are related. We conducted measurements in the presence of 20 µM aspirin which, as shown in Figure 1, completely ablates early PG production by the macrophages. Figure 5B shows that the presence of aspirin increased *Tnf* gene induction by 2-fold. Importantly, if the incubations received exogenous PGE_2_ (0.5–1 µM) to overcome the inhibition of COX-1 by aspirin, the *Tnf* induction levels dose-dependently decreased to reach the levels found in the absence of aspirin (Figure 5B). These data suggest that PGE_2_ produced by COX-1 during the early steps of macrophage activation restricts TNFα production in macrophages.

## 4. Discussion

Macrophages are known to respond to a wide variety of stimuli by mobilizing free AA and efficiently converting it to a number of oxygenated products with key roles in inflammation [4,5]. In this work we describe the early generation of PG by mouse peritoneal macrophages, a process involving COX-1, and how induction of the *Tnf* gene is influenced by this event. The immediate PG production by activated peritoneal macrophages is shown to involve cPLA_2_α activation to provide free AA, which is known to constitute a limiting factor for eicosanoid production [63,64,65].

For our studies, we have utilized yeast-derived zymosan to activate the cells. Zymosan has been widely used for many years to investigate the phospholipase A_2_-dependent pathways for lipid mediator production in murine peritoneal macrophages [66,67,68,69,70]. These cells have been found to contain high amounts of AA in membrane phospholipids; 20–25% of total fatty acid content is AA [71,72]. Importantly, macrophages also exhibit a characteristic distribution of AA among phospholipids, with PE, not PC, constituting the richest AA containing class, and PI containing much lesser amounts than PE or PC [18,21]. Among molecular species, the ethanolamine plasmalogens are markedly enriched with AA [56,73]. Further, AA does not distribute uniformly among membranes. Rather, specific phospholipid pools appear to exist that may accumulate AA at specific membrane locations within the cell [57,74]. There is now abundant evidence that the multiple phospholipase A_2_s present in cells mobilize AA for eicosanoid biosynthesis with different spatial and temporal characteristics [75]. Thus, not all cellular AA pools may be accessible to the same phospholipases. Hence AA compartmentalization may also constitute another limiting factor for eicosanoid biosynthesis. 

Given this asymmetric distribution of AA in cells, in this work we considered the intriguing possibility that, depending on the phospholipid source of free AA, certain eicosanoids could be produced in preference over others. We explored this point by taking advantage of the fact that the incorporation and distribution of AA between cellular AA pools greatly depends upon the time of incubation of the cells with the fatty acid. At short incubation times (up to 2 h), the phospholipid classes that incorporate most of the exogenous fatty acid are PC and PI, whereas PE is labeled more slowly [60,76]. However, at long incubation times (>6 h), PE becomes the major AA-containing class due to the continuing transfer of AA moieties from PC to PE via CoA-independent transacylation reactions [60,76]. Thus, by labeling the cells with [^14^C]AA at short incubation times and [^3^H]AA at long incubation times, we could establish a different ^3^H/^14^C ratio for each phospholipid class [50,77]. 

By comparing the ^3^H/^14^C ratios of PGE_2_ and 6-keto-PGF_1α_ with those of the various phospholipid classes, it was possible to establish precursor/product relationships. Although the interpretation of these data may be complicated by the phenomenon of mixing AA pools as well as the molecular heterogeneity of each phospholipid class [50], some definite conclusions can be drawn. The finding that the ^3^H/^14^C ratio for PGE_2_ is almost identical to that of PC strongly suggests that this PG arises mostly, if not exclusively, from the AA liberated from PC. As for 6-keto-PGF_1α_, the stable product of PGI_2_, its ^3^H/^14^C ratio is intermediate between those of PC and PI, suggesting that both of these pools participate in the process, albeit a contribution of PE, if minor, cannot be ruled out. 

In mouse resident peritoneal macrophages, PE constitutes the major AA-containing class (45–50% of total cellular AA), followed by PC (35–40%), and PI (5–15%) [18,21]. Note that the ^3^H/^14^C ratio for extracellular free AA is considerably higher than that of the prostaglandins, thus suggesting a substantial contribution of PE, in addition to PC and PI, to AA release. Because cPLA_2_α is responsible for AA mobilization under these conditions, the participation of all kinds of phospholipids to overall AA release is fully consistent with the view that this enzyme does not distinguish among phospholipid head groups [78].

We stress that our double-labeling approach does not allow to quantify the relative contribution of each phospholipid class to overall AA release, which could be highly dependent on the absolute amounts of AA in each phospholipid class and their cellular compartmentalization. Collectively however, the data highlight the central role that PC plays in PG production, thus providing strong support to the concept that specific phospholipid pools are linked to the formation of specific eicosanoids. These conclusions are consistent with previous work in neutrophils [79] and macrophages [15] suggesting that PC molecular species are major donors of the AA used for the formation of lipoxygenase products, and also with comprehensive lipidomic studies in macrophages [14] suggesting as well the importance of PC as a major AA source for eicosanoid biosynthesis. Importantly, our work also highlights another striking function for AA-linked PC in activated cells, i.e., to donate AA moieties to replenish the fatty acid that is lost from PE species via direct transacylation. While the physiological and/or pathophysiological consequences of such transfer are yet to be fully established, it seems likely that the reaction may constitute an important point of control of the whole eicosanoid response, because diverting AA from PC to PE prevents the fatty acid from being used by cPLA_2_α to feed COX-1 for immediate PG production. The relative importance of these two AA fluxes arising from PC, i.e., cPLA_2_α-mediated release of AA versus direct channeling to PE, may be determined by the nature of stimulus and activation conditions. 

The direct transacylation of AA moieties from PC to PE explains well our finding that, although PE contributes to early AA mobilization, the overall amount of AA in this phospholipid class remains relatively constant. The finding that the AA released from PE appears not to contribute significantly to early PG production by the activated macrophages was unanticipated. However, this result would be fully consistent with the view that maintaining high quantities of AA within PE phospholipid species may not be necessarily related to regulatory aspects of AA homeostasis and eicosanoid metabolism but to biophysical effects and interactions of AA-containing PE molecules with other membrane components to sustain different biological responses [80]. We have recently shown that the plasmalogen subclass of AA-containing PE participates in the execution of certain macrophage response such as bacterial lipopolysaccharide priming [20] or phagocytosis [17], but not others such as eicosanoid synthesis (this study). As discussed elsewhere [81,82], the relative content of AA-containing PE, especially the plasmalogen subclass, which is frequently found in lipid rafts [83,84,85], may affect key cellular properties such as fluidity, tendency to fusion, packing, thickness, and density, thereby influencing membrane transport and transmembrane signaling. Moreover, recent research has placed certain AA-containing PE phospholipid species at the center of the cellular machinery leading to ferroptotic cell death [86,87]. The relevance of some of these phospholipid species to neurodegenerative and neurodevelopmental disorders has also been emphasized [88]. It is conceivable that the AA deacylation/reacylation processes that PE molecular species are engaged in during cellular activation may be related to remodeling processes that are needed to place particular AA-containing PE species in the appropriate cellular compartments. This would lead to interaction with specific cellular components that lead to the execution of specific responses.

The other striking feature of the present work is the finding that early PG production modulates the extent of TNFα induction. Macrophages possess the ability to generate significant PG amounts immediately after exposure to stimuli, as shown in the present study. This effect has been attributed to the relatively high level of COX-1 expression of macrophages compared to many other cell types [47]. Our results agree with this view because pharmacological inhibition of COX-1, but not COX-2, almost completely abolishes PG production. Importantly, *Tnf* induction is markedly elevated when COX-1 is inhibited, and the effect can be reversed by addition of exogenous PGE_2_ to the incubation media. These results highlight a direct relationship between the two responses. While exploring the specific target(s) within the molecular machinery of *Tnf* induction through which prostaglandins exert their effect falls outside of the scope of the present work, we speculate that, in analogy with other studies [89,90], prostaglandins, acting through their specific receptors, may increase the intracellular cAMP concentration, which in turn leads to reduced gene expression. Exploring this issue will be the focus of future work from our laboratory. The finding that the early induction of cytokines such as TNFα is negatively regulated by prostaglandins may have a significant impact on the overall inflammatory response of the macrophages to immune innate stimuli, and provides a pharmacological target to manipulate the extent of such response. In turn, since the PG response depends on the supply of free AA by cPLA_2_α activation, our results also highlight the key role that this enzyme plays in the early macrophage responses to microbial infection that help modulate the expression of genes involved in the inflammatory response.

## 5. Conclusions

The possibility that distinct AA-containing glycerophospholipid pools, including the plasmalogens, are used by inflammatory stimuli to elicit a specific eicosanoid response, constitutes an intriguing current line of research. In this paper we contribute to strengthening this concept by showing that PC is a key source of AA used for the immediate synthesis of PGE_2_, and likely also of PGI_2_. Thus the compartmentalized distribution of AA among membrane phospholipid classes may constitute an effective means to regulate both the levels and nature of eicosanoids produced under different stimulatory conditions. Furthermore, our data also reinforce the important role that cPLA_2_α and COX-1 play in the innate inflammatory response by modulating the early expression of genes such as *Tnf*, thus contributing to limit inflammation. Altogether, these results provide novel information to increase our understanding of the cellular pathways that regulate AA bioavailability, and its subsequent conversion to eicosanoids. In addition, the data help to improve our understanding of the host defense to fungal aggression, which may facilitate the development of therapies to treat these challenging and difficult to treat disorders.

## Data Availability

Data are contained within the article or supplementary material.

## Abbreviations

AA	arachidonic acid
COX	cyclooxygenase
GC/MS	gas chromatography coupled to mass spectrometry
LC/MS	liquid chromatography coupled to mass spectrometry
cPLA_2_α	group IVA cytosolic phospholipase A_2_
iPLA_2_β	group VIA calcium-independent phospholipase A_2_
PC	choline-containing glycerophospholipids
PE	ethanolamine-containing glycerophospholipids
PI	phosphatidylinositol
PG	prostaglandin
TNFα	tumor necrosis factor-α
